# Transcriptome Analysis of Orbital Adipose Tissue in Active Thyroid Eye Disease Using Next Generation RNA Sequencing Technology

**DOI:** 10.2174/1874364101812010041

**Published:** 2018-04-16

**Authors:** Bradford W. Lee, Virender B. Kumar, Pooja Biswas, Audrey C. Ko, Ramzi M. Alameddine, David B. Granet, Radha Ayyagari, Don O. Kikkawa, Bobby S. Korn

**Affiliations:** 1 Department of Ophthalmology, Division of Ophthalmic Plastic and Reconstructive Surgery, University of California, San Diego, La Jolla, CA; 2 Department of Ophthalmology, University of California, San Diego, La Jolla, CA; 3 Division of Plastic Surgery, Department of Surgery, University of California, San Diego, La Jolla, CA; 4 Division of Oculofacial Plastic and Reconstructive Surgery, Bascom Palmer Eye Institute, University of Miami Miller School of Medicine, Miami, FL

**Keywords:** Orbital adipose tissue, Thyroid eye disease, Transcriptome, Next generation sequencing, RNA sequencing technology, IGF-1 signaling

## Abstract

**Objective:**

This study utilized Next Generation Sequencing (NGS) to identify differentially expressed transcripts in orbital adipose tissue from patients with active Thyroid Eye Disease (TED) *versus* healthy controls.

**Method:**

This prospective, case-control study enrolled three patients with severe, active thyroid eye disease undergoing orbital decompression, and three healthy controls undergoing routine eyelid surgery with removal of orbital fat. RNA Sequencing (RNA-Seq) was performed on freshly obtained orbital adipose tissue from study patients to analyze the transcriptome. Bioinformatics analysis was performed to determine pathways and processes enriched for the differential expression profile. Quantitative Reverse Transcriptase-Polymerase Chain Reaction (qRT-PCR) was performed to validate the differential expression of selected genes identified by RNA-Seq.

**Results:**

RNA-Seq identified 328 differentially expressed genes associated with active thyroid eye disease, many of which were responsible for mediating inflammation, cytokine signaling, adipogenesis, IGF-1 signaling, and glycosaminoglycan binding. The IL-5 and chemokine signaling pathways were highly enriched, and very-low-density-lipoprotein receptor activity and statin medications were implicated as having a potential role in TED.

**Conclusion:**

This study is the first to use RNA-Seq technology to elucidate differential gene expression associated with active, severe TED. This study suggests a transcriptional basis for the role of statins in modulating differentially expressed genes that mediate the pathogenesis of thyroid eye disease. Furthermore, the identification of genes with altered levels of expression in active, severe TED may inform the molecular pathways central to this clinical phenotype and guide the development of novel therapeutic agents.

## 1 INTRODUCTION

Thyroid Eye Disease (TED) is caused by a systemic autoimmune attack on the orbit and other target tissues, including the thyroid, skin, and pretibial soft tissues [1]. Circulating lymphocytes and humoral agents infiltrate the orbital soft tissues and induce orbital fibroblasts to cause the characteristic pathological changes of TED, such as orbital adipose tissue expansion, muscle fibrosis, and deposition of glycosaminoglycans within the extraocular muscles [2, 3]. Various molecular factors have been implicated in TED pathogenesis, including insulin like growth factor-1 and interleukins [4], although most studies have relied on cultured cell lines from TED patients [5].

TED follows a stereotypical disease course (“Rundle’s curve”) consisting of an “active phase,” characterized by inflammation and dynamically worsening orbitopathy, followed by a “quiescent phase” of disease stability. About 3-5% of patients develop severe TED associated with vision loss and compressive optic neuropathy [6]. Treatments for active TED include systemic corticosteroids, orbital radiation, biological immunomodulatory agents, and induction of euthyroid status, in some cases by thyroidectomy. Previous studies have used microarray technology to study differential gene expression in orbital fat in TED and have identified Wnt signaling genes, adipocyte-related immediate early genes, and IGF-1 signaling genes as being potentially implicated in pathogenesis [7-10]. Other in vitro studies on orbital adipose-derived stem cells harvested from patients with TED used RNA Seq and found downregulation of early neural crest markers and ectopic expression of *HOX* genes [11]. This study aimed to characterize the RNA transcriptome in the orbital adipose tissue of patients with severe, active TED compared to that of matched, healthy controls. We utilized Next Generation Sequencing (NGS) to identify differential gene expression patterns and potential therapeutic targets for translational research and prospective clinical trials.

## 2 METHODS

### 2.1 Study Design

This prospective case-control study was approved by the University of California, San Diego Institutional Review Board, and was performed in accordance with the Declaration of Helsinki. Study cases all had severely affected phenotypes of TED in the active phase of the disease when orbital decompression surgery was performed Fig. (**1**). All cases had actively worsening signs and symptoms, and two had compressive optic neuropathy while the third had a Thyroid Stimulating Immunoglobulin (TSI) level > 500. Clinical Activity Score (CAS) was determined by a board-certified ophthalmologist and oculoplastic surgeon based on history and examination findings^1^. Two cases had CAS scores of 7 and one had a CAS score of 8 on a scale with a maximum score of 10. Controls had dermatochalasis and no history of thyroid abnormalities or TED.

Inclusion and exclusion criteria were strict to control for confounding factors suspected of altering transcriptional activity in orbital adipose tissue in TED or normal controls. All cases and controls were Caucasian and female to control for gender and racial transcriptional variations. Any patients with significant current or recent tobacco smoking history were excluded, since smoking is well known to increase the incidence and severity of TED and induce numerous gene expression changes [3]. Factors that influence adipogenesis and inflammation were also controlled. Patients were excluded if they were overweight or obese (BMI over 25 kg/m^2^), had diabetes or metabolic syndrome, or were currently or recently taking any systemic steroids, immunomodulatory agents, or had undergone orbital radiation. All controls had no major medical problems, history of thyroid abnormalities or clinical evidence of TED. For detailed clinical characteristics, see Table (**1**).

### 2.2 Orbital Adipose Tissue for Transcriptome Analysis

Orbital adipose tissue was harvested from cases and controls at the time of orbital decompression and blepharoplasty, respectively, and typically consisted of approximately 1-2 mL of tissue. Similar orbital adipose tissue depots were selected to ensure an unbiased representation. Adipose tissue was immediately placed in specimen tubes on dry ice and directly transferred for storage at -86 degrees Celsius.

### 2.3 RNA Sequencing and Identification of Differentially Expressed Genes

Total RNA was isolated using the RNeasy Mini Kit (Qiagen, Valencia, CA) according to the manufacturer’s protocol. The RNA concentration and purity were verified spectrophotometrically using the 260/280 ratio and were found to be within the 1.8-2.2 range required for RNA-Seq experiments. Samples were converted into Tru-Seq libraries for sequencing on the Illumina HiSeq2000 platform (Illumina, San Diego, CA) at the university’s RNA-Seq core research facility. The total amount of RNA for each sample was ≥ 5 µg with a concentration ≥ 80 ng/µl in nuclease-free water. Output data was analyzed by a professional senior bioinformatics engineer at the UCSD Center for Computational Biology and Bioinformatics. Ribosomal RNA filtering was performed using Array Studio NGS analytics (http://www.omicsoft.com/array-studio) and demonstrated successful ribosomal RNA depletion with only 1.5 to 3.8% rRNA sequences filtered. The Array Studio Raw Data QC Wizard was used to examine the reads that passed filtering for each sample and found that all samples had at least 30 million reads. Filtered reads alignment also demonstrated over 80 million paired, uniquely mapped reads for all samples. Gene expression of transcripts was calculated with the measurement of counts and RPKM (Reads Per Kilobase of transcript per Million mapped reads) using the Array Studio Report Gene/Transcript Counts functionality. Filtering was then performed to select for a False Discovery Rate (FDR) adjusted p-value < 0.05 using the Benjamini-Hochberg method.

### 2.4 Analysis of Transcripts

To detect molecular functions, biological processes, and pathways associated with the differential expression signature, Gene Ontology (GO, http://www.geneontology.org) analysis was performed using ToppGene (https://toppgene.cchmc.org/enrichment.jsp). Additional databases utilized included: Kyoto Encyclopedia of Genes and Genomes (http://www.genome.jp/kegg/pathway.html), WikiPathways (www.wikipathways.org), Reactome (http://www.reactome.org), Comparative Toxicogenomics Database (http://ctdbase.org), STITCH (http://stitch.embl.de), and Broad Institute Connectivity Map (https://www.broadinstitute.org/cmap/).

### 2.5 Validation of Expression Levels of Selected Genes by Quantitative Reverse Transcription-Polymerase Chain Reaction (qRT-PCR)

Total RNA was isolated from all patient samples using RNeasy Mini Kit (Qiagen, CA, USA). The primers were designed for qRT-PCR using Primer3. cDNA synthesis was performed using the standard protocol of BioRad (iScript cDNA Synthesis Kit, USA). qRT-PCR was performed in duplicate for each of the cases and controls for various top-ranked differentially expressed genes of interest: CCL2, S100A9, VCAN, and SERPINA1. Analysis of gene expression relative to the housekeeping gene ACTIN was performed as previously described.^2^ A Student’s T-test was performed to determine p-values and statistical significance between cases and an average of the three controls.

## 3 RESULT

### 3.1 Differentially Expressed Genes Between Severe, Active TED Patients and Healthy Controls

RNA-Seq yielded a total of 57,736 genes tested, and 352 genes were identified having adjusted p-values < 0.05 after excluding outliers. After filtering for a minimum read count of 5 for all cases and controls, 328 genes comprised the final differential expression signature of which 52 were downregulated and 276 were upregulated relative to controls Fig. (**2**). There were 44 genes with very low FDRs (< 0.0001), including 5 downregulated genes (-2.3 to -4.3 fold change) and 39 upregulated genes (+2.0 to +8.8 fold change) Table (**2**).

### 3.2 Enriched Functions, Biological Processes, and Pathways of the Differential Expression Signature

Analysis of the differential expression signature showed enrichment for cell adhesion and small molecule binding functions, including receptor binding, fibronectin binding, cell adhesion molecule binding, immunoglobulin receptor binding, receptor activity, and molecular transducer activity. Of particular relevance to TED, lipid binding and glycosaminoglycan binding were among the most enriched functions. Additionally, very-low-density lipoprotein particle receptor activity was also enriched with two differentially expressed genes from the signature out of 4 genes in the annotation (p = 1.37E-03, FDR B&H = 2.80E-02).

Biological processes enrichment analysis showed a large predominance of immune response and leukocyte activation and migration pathways, such as immune response, regulation of immune system process, positive regulation of immune system response, leukocyte activation and migration, and granulocyte migration Table (**3**).

Pathway enrichment analysis showed that two of the top four ranked pathways were pro-inflammatory cytokine pathways: the chemokine signaling pathway (p = 7.72E-10, FDR B&H = 3.30E-07) and the IL-5 Signaling Pathway (p=7.82E-09, FDR B&H = 2.51E-06), which contained twenty and twelve genes, respectively Table (**4**).

### 3.3 Drugs Enriched for the Differential Expression Signature

Drugs with genes most highly enriched in the signature included tobacco smoke (FDR B&H: 3.17E-16); retinoic acid-related compounds like tamibarotene, isotretinoin, and retinoic acid (FDR B&H from 3.17E-16 to 1.21E-08); 8-isoprostaglandins E1 and E2 (FDR B&H <5.19E-09); and simvastatin (FDR B&H: 1.12E-10) Table (**5**).

### 3.4 qRT-PCR Analysis of CCL2, S100A9, VCAN, and SERPINA1

Four upregulated genes were selected among the top-ranked differentially expressed genes in the signature for validation by qRT-PCR. S100A9 showed a 9.40-fold increased expression (p < 0.0001), CCL2 showed a 7.25-fold increased expression (p < 0.0001), VCAN showed a 1.68-fold increased expression (p = 0.0018), and SERPINA1 showed a 1.24-fold increased expression (p = 0.0811) among the cases group relative to the controls Fig. (**3**).

## 4 DISCUSSION

In this prospective case-control study, we sought to identify differences in the transcriptome between active, severe TED patients and healthy controls using NGS. Cases with active rather than quiescent TED were selected to ensure that differential gene expression findings were associated with an actively worsening clinical phenotype of TED. This study selected severe TED cases with a CAS of 7-8 and actively worsening signs and symptoms, with the goal of maximizing the yield of differentially expressed genes relevant to TED.

Understanding the pathogenesis of TED has been greatly facilitated by the study of cultured orbital fibroblasts from TED patients [12]. Previously published studies utilized the “candidate” gene approach to identify differential gene expression using cultured cell models and have identified multiple transcripts related to the inflammatory cascade [13]. These studies created primary cell cultures of orbital fibroblasts isolated from orbital adipose tissue, but the validity of such experiments conducted in tissue culture media without the in vivo environment and intact immune system remains unclear. Linquist et al studied fresh orbital tissues to assay gene expression for eight pre-selected cytokines [14]. While more physiological, this study was limited by the candidate gene approach, since a limited number of pre-selected transcripts were assayed. Moreover, the study included patients in the quiescent stage of TED with no active inflammation.

To enhance the validity of our findings, we eliminated confounding factors, such as tobacco smoking, use of systemic steroids or immunomodulatory medications, and orbital radiation therapy, all of which modulate the immune system and likely alter the transcription of genes involved in TED pathogenesis [2]. Previous studies have not controlled for these confounding factors [7, 8, 11, 14] or did not provide these data [9, 10]. We chose cases and controls of the same gender and racial ancestry and excluded potential subjects who were overweight or obese-due to their tendency toward systemic increased adipogenesis—or who had diabetes or metabolic syndrome. Diabetes medications like thiazolidinediones are known to activate Peroxisome Proliferator-Activated Receptors (PPARs), which have been linked to thyroid eye disease on a clinical basis as well as in previously published differential gene expression studies [9, 15, 16]. Not surprisingly, rosiglitazone, a thiazolidinedione, was found to be highly linked to differentially expressed genes in our study Table (**5**).

This is also the first study to use NGS technology in the form of RNA-Seq using the Illumina HiSeq2000 platform on in vivo orbital adipose tissue in TED. RNA-Seq provides a broader dynamic range, since unlike microarray technology, its gene expression measurement is not limited by background at the low end and signal saturation at the high end. It is able to yield absolute rather than relative expression values, and it offers superior sensitivity and specificity in differential expression studies [17].

Perhaps due to the central role of orbital fat expansion in TED, previous studies have focused on Wnt signaling genes [7, 9, 11], which regulate adipogenesis, as well as the IGF-1 pathway genes [9], which are implicated in adipogenesis and induce HA synthesis. Other studies have focused on upregulation of the immediate early genes, which are induced during the initial proliferative phase of preadipocytes and other adipogenesis-related genes [7, 8, 10]. Our study found several genes in the differential expression signature that were reported in prior studies, such as IGF1 (insulin-like growth factor 1), FADS1 (fatty acid desaturase 1), and immediate early genes BTG2 (BTG anti-proliferation factor 2). Moreover, 26 genes in the differential expression signature were involved in lipid binding and 12 genes were involved in glycosaminoglycan binding. However, our study selected different types of cases and controls from prior studies and looked broadly at the differential expression signature for enriched pathways and functions.

Among the biological functions and pathways enriched in the differential expression signature, the top functions and processes expectedly included immunoglobulin binding, leukocyte activation and migration, cell adhesion and small molecule binding, and various other immune-regulated functions. Previous studies have documented specific cytokine-dependent fibroblast activation [18]. IFN-gamma, TNF-alpha, and IL-1-alpha have been detected in orbital fat from patients with TED [19], and cytokines such as IL-4, IL-6, and IL-10 have been detected in affected extraocular muscle and orbital fat [20].

In addition to specifically identifying 20 genes involved in the chemokine pathway that were upregulated Table (**4**), our enrichment analysis identified the IL-5 signaling pathway as among the most enriched pathways with 12 upregulated genes identified Table (**4**). IL-5 is a cytokine produced by type-2 T helper cells and mast cells, is involved in stimulating B cell growth and immunoglobulin secretion, and is a key mediator of eosinophil production and activation [21]. Mepolizumab and reslizumab are IL-5 inhibitors that are FDA-approved for severe refractory asthma and eosinophil-mediated inflammation [22, 23]. Given the predominance of this pathway, these medications deserve further study for their potential role in treating TED.

Our analysis also demonstrated marked enrichment for the very-Low-Density Lipoprotein (vLDL) receptor activity function, with apolipoprotein B receptor and low density lipoprotein receptor both showing significant upregulation. Orbital adipogenesis is central to TED, and upregulation of the LDL receptor allows increased cholesterol and fatty acid uptake by cells that is necessary for lipid storage, cell division, and production of cholesterol-derived steroid hormones [24]. The Sterol-Regulatory Element Binding Protein (SREBP) pathway is the master regulator of the lipid biosynthetic pathway and mediates the increase in lipid biosynthesis [25]. Post-translational regulation of lipid biosynthesis would not be captured by RNA-Seq analysis, and follow-up functional studies at the protein level are planned to confirm these initial RNA-Seq findings.

Our enrichment analysis showed that simvastatin was linked to 35 genes in the signature, while atherosclerosis was linked with 6 genes in the signature Table (**5**). This finding is even more significant as none of our patients were taking hydroxymethylglutaryl-CoA reductase inhibitors (statins), which are known to upregulate the LDL receptor [24]. Statins have been shown to improve cholesterol levels, reduce cardiovascular events, and have anti-inflammatory actions independent of their cholesterol-lowering role [26]. A large, longitudinal cohort study of patients with Graves’ disease found that statin use was associated with reduced incidence of TED, although no similar associations were found for non-statin cholesterol lowering medications [27]. Given this epidemiological association and our preliminary gene expression data showing upregulation of APOBR and LDLR in TED and statins’ links to numerous upregulated genes in the signature, further molecular studies and clinical trials are warranted to evaluate the possible therapeutic benefits of statin therapy in TED.

The primary limitation of our study is its small sample size. The stringent inclusion and exclusion criteria for cases and controls resulted in limited patients eligible for the study. While the qRT-PCR data largely support the RNA-Seq data for differentially expressed genes, a greater sample of cases and controls for qRT-PCR experiments would further confirm the differential expression of genes identified in this study. Despite a small sample size, this study used NGS technology and obtained results with robust p-values and false discovery rates. Moreover, the prospective case-control study design and exclusion of potential confounders affecting gene expression enhance the validity of this study’s findings.

Another limitation of this study is that cases selected for our study had predominantly Type II TED with marked extraocular muscle enlargement. Thus, the differential expression signature in this study may not apply for milder cases, quiescent disease, or Type I TED cases mainly affected by orbital fat enlargement.

Finally, the value of RNA-Seq studies lies in their ability to generate hypotheses and candidate genes and pathways for further studies. While the qRT-PCR validation studies largely support the RNA-Seq data for differentially expressed genes, further studies with larger samples of cases and controls are needed to test the hypotheses generated from this study via qRT-PCR and Western Blot experiments. They might provide a more definitive answer on the involvement or lack of involvement of genes in the differential expression signature implicated in TED.

## CONCLUSION

In conclusion, this study is the first prospective case-control study to investigate differential gene expression in orbital adipose tissue from active, severe TED patients and healthy controls. It is also the first to use RNA-Seq technology in profiling the transcriptome. The differential expression signature and enrichment analysis were concordant with prior differential expression studies and identified numerous genes involved in inflammation, cytokine signaling, adipogenesis, the IGF-1 signaling pathway, and glycosaminoglycan binding. Notably, it identified vLDL receptor activity, the LDL receptor, and the Apolipoprotein B receptor along with statins as being implicated in TED, thereby providing a molecular corroboration of epidemiological data linking statin used to reduced incidence of TED.^49^ Finally, while our study findings are biologically plausible with robust statistical significance, larger confirmatory studies are needed with further exploration of the idiosyncrasies and diverse presentations of TED.

## Figures and Tables

**Fig. (1):**
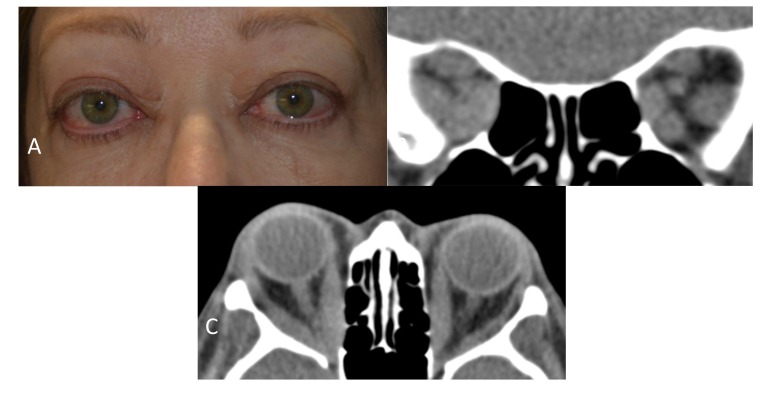
**A:** Clinical photograph of a case with severe, active thyroid eye disease with exophthalmos, congestive orbitopathy, and compressive optic neuropathy. **B:** A Computed Tomography (CT) coronal section through the orbital apex shows enlargement of the extraocular muscles and compression of the optic nerve on the right side. **C:** A CT axial section demonstrates marked enlargement of the extraocular muscles with right optic nerve compression at the orbital apex.

**Fig. (2):**
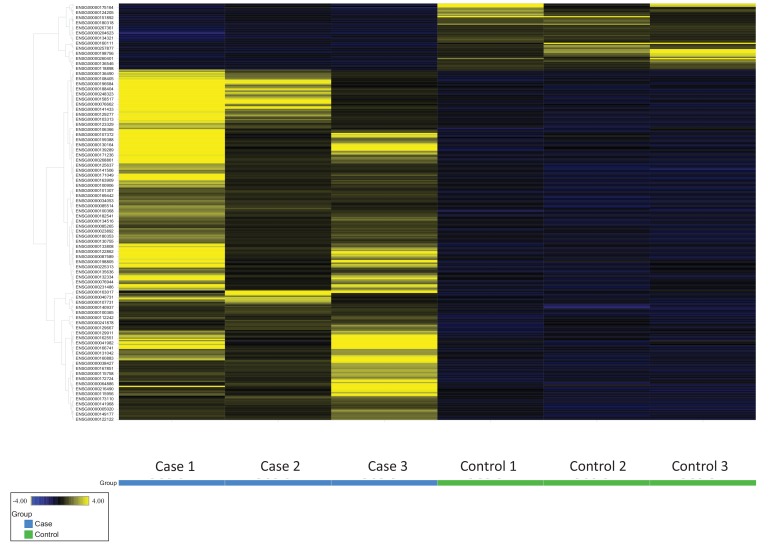
Heat map of clustering analysis of upregulated and downregulated genes in TED cases compared to healthy controls. Individual cases and controls are listed along the X-axis, and differentially expressed genes are listed along the Y-axis.

**Fig. (3):**
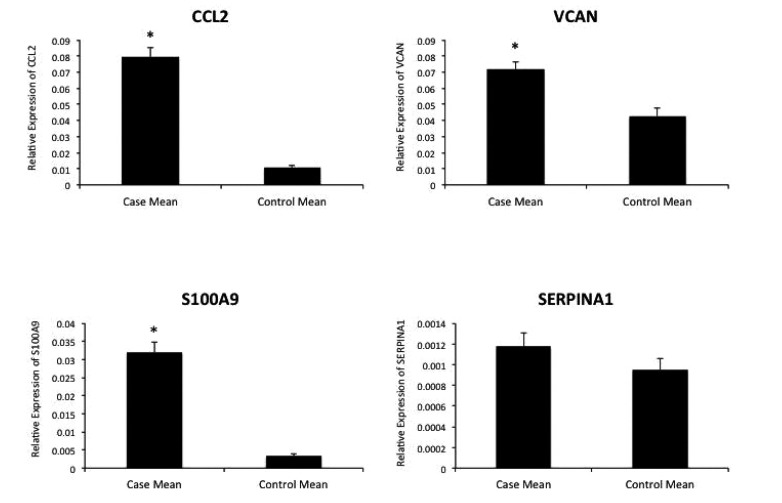
Reverse transcription PCR results from differentially expressed genes CCL2, VCAN, S100A9, and SERPINA1 in cases and controls. The asterisk (*) indicates p < 0.05.

**Table 1:** Clinical characteristics of cases undergoing orbital decompression and controls undergoing blepharoplasty.

-	**Case 1**		**Case 2**	**Case 3**		**Control 1**	**Control 2**		**Control 3**
**Age (years)**	68		81	62		60	58		80
**Gender**	F		F	F		F	F		F
**Race**	Caucasian		Caucasian	Caucasian		Caucasian	Caucasian		Caucasian
**Duration of Grave's disease prior to surgery (mo)**	12		96	20		N/A	N/A		N/A
**Duration of TED prior to surgery (mo)**	6		8	20		N/A	N/A		N/A
**Previous treatment for Grave's disease**	Methimazole, radioactive iodine		Methimazole	Methimazole		N/A	N/A		N/A
**Previous treatment for TED**	Selenium supplements		Peribulbar steroid injection to OS, fat specimen from OD	Selenium supplements		N/A	N/A		N/A
**Smoking history**	10 pack-years, quit 40 years prior		7.5 pack-years, quit 44 years prior	0.2 pack-years, quit 3 years prior		None	None		None
**Body mass index (kg per m^2)**	22.9		24.2	24.1		22.7	22.8		22.3
**Exophthalmometry, Naugle (mm)**	24.5 OD, 24.5 OS		26 OD, 30 OS	23 OD, 22 OS		N/A	N/A		N/A
**Thyroid stimulating immunoglobulin (TSI) level at time of surgery**	Not available		>500	530		N/A	N/A		N/A
**Presence of compressive optic neuropathy**	Yes		No	Yes		No	No		No
**CAS Score (Range 0-10)**	7		7	8		N/A	N/A		N/A

“Pack-years” refers to the number of packs of cigarettes smoked per day multiplied by the number of years a person has smoked.

**Table 2:** Top 44 differentially expressed genes in orbital adipose tissue in severe, active TED.

**Gene Symbol**	**Gene Description**	**Fold Change**	**P Value**	**FDR_BH**
Downregulated Genes				
MASP1	mannan-binding lectin serine peptidase 1 (C4/C2 activating component of Ra-reactive factor]	-4.18	1.46E-10	3.90E-07
ALX1	ALX homeobox 1	-3.16	2.74E-10	6.03E-07
LOC1004211	SEC24 family member A pseudogene	-2.26	2.71E-10	6.03E-07
LINC01139	long intergenic non-proiein coding RNA 1139	-4.29	2.55E-09	4.68E-06
LGR5	leucine-rich repeat containing G protein-coupled receptor 5	-3.93	2.28E-08	2.31E-05
Upregulated Genes				
PKD1P5	polycystic kidney disease 1 (autosomal dominant) pseudogene 5	5.50	3.31E-29	1.24E-24
S100A9	S100 calcium binding protein A9	8.82	7.40E-19	1.39E-14
SIRPB1	signal-regulatory protein beta 1	6.09	1.43E-15	1.78E-11
HSPA6	heat shock 70kDa protein 6 (HSP70B')	3.89	4.05E-15	3.79E-11
HCK	hemopoietic cell kinase	3.72	1.53E-14	1.15E-10
FPR2	formyl peptide receptor 2	6.47	1.28E-13	7.99E-10
ALOX5	arachidonate 5-lipoxygenase	4.18	3.63E-13	1.94E-09
SERPINA1	serpin peptidase inhibitor, clade A (alpha-1 antiproteinase, antitrypsin), member 1	5.37	6.35E-12	2.64E-08
FPR1	formyl peptide receptor 1	5.48	1.26E-11	4.70E-08
RUBCNL	RUN and cysteine rich domain containing beclin 1 interacting protein like	3.03	4.88E-11	1.52E-07
LRRC25	leucine rich repeat containing 25	3.48	9.73E-11	2.80E-07
HBG1	hemoglobin, gamma A	4.99	2.12E-10	5.29E-07
TGFA	transforming growth factor, alpha	3.42	1.72E-09	3.58E-06
VCAN	versican	3.06	2.75E-09	4.68E-06
CD300A	CD300a molecule	3.37	2.73E-09	4.68E-06
CD52	CD52 molecule	3.41	2.40E-09	4.68E-06
CORO1A	coronin, actin binding protein, 1A	3.19	3.27E-09	5.32E-06
SASH3	SAM and SH3 domain containing 3	2.99	5.09E-09	7.64E-06
PTPN6	protein tyrosine phosphatase, non-receptor type 6	2.40	7.95E-09	1.15E-05
RPS6KA1	ribosomal protein S6 kinase, 90kDa, polypeptide 1	3.06	8.94E-09	1.24E-05
EMR2	egf-like module containing, mucin-like, hormone receptor-like 2	3.48	9.40E-09	1.26E-05
LILRA5	leukocyte immunoglobulin like receptor A5	4.14	1.07E-08	1.38E-05
IGHG1	immunoglobulin heavy constant gamma 1 (G1m marker)	3.52	1.31E-08	1.64E-05
LCP1	lymphocyte cytosolic protein 1 (L-plastin)	3.71	1.38E-08	1.67E-05
CCL2	chemokine (C-C motif) ligand 2	3.83	1.50E-08	1.76E-05
MEFV	Mediterranean fever	4.00	1.66E-08	1.88E-05
CYTIP	cytohesin 1 interacting protein	4.18	1.77E-08	1.95E-05
ITGAX	integrin, alpha X (complement component 3 receptor 4 subunit)	3.95	2.08E-08	2.23E-05
NCF4	neutrophil cytosolic factor 4, 40kDa	2.23	2.15E-08	2.23E-05
SAMSN1	SAM domain, SH3 domain and nuclear localization signals 1	3.91	2.56E-08	2.52E-05
ACTA2-AS1	ACTA2 antisense RNA 1	2.46	4.29E-08	4.12E-05
LYN	LYN proto-oncogene, Src family tyrosine kinase	2.82	7.13E-08	6.68E-05
DDIT4	DNA-damage-inducible transcript 4	2.01	9.24E-08	7.95E-05
FERMT3	fermitin family member 3	2.55	9.03E-08	7.95E-05
NNMT	nicotinamide N-methyltransferase	3.56	9.04E-08	7.95E-05
FFAR2	free fatty acid receptor 2	3.82	9.50E-08	7.95E-05
MMP25	matrix metallopeptidase 25	3.88	9.55E-08	7.95E-05
RBM47	RNA binding motif protein 47	2.81	1.18E-07	9.38E-05
STXBP2	syntaxin binding protein 2	3.08	1.18E-07	9.38E-05

**Table 3:** Significantly enriched molecular functions and biological processes among the differential expression signature (top 10 ranked by FDR)

**Gene Ontology ID**	**Name**	**P-value**	**FDR B&H**	**Genes from Signature**
Molecular Functions				
GO:0005102	Receptor binding	4.03E-08	3.21E-05	53
GO:0001968	Fibronectin binding	9.49E-06	3.77E-03	6
GO:0050839	Cell adhesion molecule binding	1.54E-05	3.77E-03	13
GO:0008289	Lipid binding	2.37E-05	3.77E-03	26
GO:0098772	Molecular function regulator	2.37E-05	3.77E-03	40
GO:0034987	Immunoglobulin receptor binding	5.95E-05	7.89E-03	5
GO:0004872	Receptor activity	7.95E-05	9.04E-03	45
GO:0003823	Antigen binding	9.58E-05	9.53E-03	9
GO:0005539	Glycosaminoglycan binding	1.29E-04	1.03E-02	12
GO:0060089	Molecular transducer activity	1.30E-04	1.03E-02	50
Biological Processes				
GO:0006955	Immune response	1.16E-31	5.39E-28	99
GO:0002682	Regulation of immune system process	7.71E+31	1.80E-27	94
GO:0001775	Cell activation	8.98E-30	1.39E-26	75
GO:0006952	Defense response	2.66E-28	3.10E-25	98
GO:0002684	Positive regulation of immune system process	2.18E-27	2.03E-24	70
GO:0045321	Leukocyte activation	4.12E-26	3.20E-23	62
GO:0050776	Regulation of immune response	1.61E-24	1.07E-21	68
GO:0050900	Leukocyte migration	6.19E-24	3.60E-21	42
GO:0009611	Response to wounding	1.65E-22	8.55E-20	67
GO:0097530	Granulocyte migration	2.73E-21	1.27E-18	25

**Table 4:** Chemokine signaling pathway and IL-5 signaling pathway differentially expressed genes.

**Entrez Gene ID**	**Gene Symbol**	**Gene Description**	**Fold Change**	**P Value**	**FDR_BH**
Chemokine Signaling Pathway					
409	ARRB2	Arrestin, beta 2	1.9423	4.10E-05	0.0084
6363	CCL19	Chemokine (C-C motif) ligand 19	2.6223	6.95E-05	0.0123
6347	CCL2	Chemokine (C-C motif) ligand 2	3.8291	1.50E-08	1.76E-05
6348	CCL3	Chemokine (C-C motif) ligand 3	2.9887	1.32E-05	0.0034
6351	CCL4	Chemokine (C-C motif) ligand 4	3.2848	2.72E-06	0.0011
1236	CCR7	Chemokine (C-C motif) receptor 7	2.5541	0.0001	0.0161
1794	DOCK2	Dedicator of cytokinesis 2	2.1320	1.10E-05	0.0031
2268	FGR	FGR proto-oncogene, Src family tyrosine kinase	2.4515	0.0002	0.0271
3055	HCK	HCK proto-oncogene, Src family tyrosine kinase	3.7212	1.53E-14	1.15E-10
4067	LYN	LYN proto-oncogene, Src family tyrosine kinase	2.8208	7.13E-08	6.68E-05
653361	NCF1	Neutrophil cytosolic factor 1	2.4395	0.0004	0.0438
4792	NFKBIA	NFKB inhibitor alpha	2.0860	0.0005	0.0473
57580	PREX1	Phosphatidylinositol-3,4,5-trisphosphate-dependent Rac exchange factor 1	2.4785	2.35E-05	0.0054
5293	PIK3CD	Phosphatidylinositol-4,5-bisphosphate 3-kinase catalytic subunit delta	2.2212	1.67E-05	0.0041
23533	PIK3R5	Phosphoinositide-3-kinase regulatory subunit 5	2.2802	0.0001	0.0185
5330	PLCB2	Phospholipase C beta 2	1.9807	0.0004	0.0407
5579	PRKCB	Protein kinase C beta	3.0923	2.07E-06	0.0009
7409	VAV1	Vav guanine nucleotide exchange factor 1	2.0608	7.51E-06	0.0023
10451	VAV3	Vav guanine nucleotide exchange factor 3	2.3706	3.59E-06	0.0013
7454	WAS	Wiskott-Aldrich syndrome	2.3956	1.79E-05	0.0043
IL-5 Signaling Pathway					
240	ALOX5	Arachidonate 5-lipoxygenase	4.1782	3.63E-13	1.94E-09
1439	CSF2RB	Colony stimulating factor 2 receptor beta common subunit	2.2520	6.56E-05	0.0119
3055	HCK	HCK proto-oncogene, Src family tyrosine kinase	3.7212	1.53E-14	1.15E-10
3059	HCLS1	Hematopoietic cell-specific Lyn substrate 1	2.7164	1.13E-06	0.0006
3689	ITGB2	Integrin subunit beta 2	2.6061	7.44E-07	0.0004
3385	ICAM3	Intercellular adhesion molecule 3	2.4011	0.0004	0.0438
4067	LYN	LYN proto-oncogene, Src family tyrosine kinase	2.8208	7.13E-08	6.68E-05
4792	NFKBIA	NFKB inhibitor alpha	2.0860	0.0005	0.0473
5579	PRKCB	Protein kinase C beta	3.0923	2.07E-06	0.0009
5777	PTPN6	Protein tyrosine phosphatase, non-receptor type 6	2.3965	7.95E-09	1.15E-05
6195	RPS6KA1	Ribosomal protein S6 kinase, 90kDa, polypeptide 1	3.0582	8.94E-09	1.24E-05
7409	VAV1	Vav guanine nucleotide exchange factor 1	2.0608	7.51E-06	0.0023

**Table 5:** Diseases and drugs enriched for the differential expression signature.

**ID**	**Name**	**P Value**	**FDR B&H**	**Genes**
Diseases				
CTD:D005922	IgA glomerulonephritis	6.60E-12	4.48E-09	35
CTD:D011658	Pulmonary fibrosis	4.20E-07	1.42E-04	9
CTD:D001172	Rheumatoid arthritis	1.72E-05	3.04E-03	14
CTD:D003424	Crohn's disease	2.57E-05	3.49E-03	7
CTD:D006967	Hypersensitivity	3.99E-04	1.94E-02	7
CTD:D001171	Juvenile arthritis	4.45E-04	1.94E-02	10
CTD:D050197	Atherosclerosis	4.51E-04	1.94E-02	6
CTD:D017449	Allergic contact dermatitis	4.83E-04	1.94E-02	7
Drugs				
CTD:D014028	Tobacco smoke	6.42E-20	3.17E-16	74
CTD:C061133	Tamibarotene	6.87E-20	3.17E-16	44
CTD:D003907	Dexamethasone	3.40E-10	1.18E-15	74
Stitch:CID000003003	Betamethasone-d5	2.84E-14	3.92E-11	57
CTD:D019821	Simvastatin	1.00E-13	1.12E-10	35
CTD:D015474	Isotretinoin	3.01E-13	2.98E-10	41
CTD:D001241	Aspirin	1.17E-12	9.52E-10	37
Stitch:CID000000158	8-isoprostaglandin E2	4.58E-12	3.25E-09	34
Stitch:CID000000214	8-isoprostaglandin E1	7.88E-12	5.19E-09	22
Stitch:CID010447660	IL-1 receptor antagonist	1.48E-09	5.99E-09	13
Broad:1152 UP	Retinoic acid; Up 200	2.01E-11	1.21E-08	17
CTD:D008727	Methotrexate	1.80E-11	1.27E-08	50
CTD:C089730	Rosiglitazone	2.07E-10	1.03E-07	50

## Footnotes

1 Methodology for determining CAS score can be found in the following reference: Mourits MP, Koornneef L, Wiersinga WM, Prummel MF, Berghout A, van der Gaag R. Br J Ophthalmol. 1989 Aug;73(8):639-44.

2 For detailed description of the protocol, refer to reference: Mandal MN, Ambasudhan R, Wong PW, et al. Characterization of mouse orthologue of ELOVL4: genomic organization and spatial and temporal expression. Genomics. 2004 Apr;83(4):626-35.
